# A probiotic complex, rosavin, zinc, and prebiotics ameliorate intestinal inflammation in an acute colitis mouse model

**DOI:** 10.1186/s12967-018-1410-1

**Published:** 2018-02-21

**Authors:** Jin-Sil Park, JeongWon Choi, Ji Ye Kwon, Kyung-Ah Jung, Chul Woo Yang, Sung-Hwan Park, Mi-La Cho

**Affiliations:** 1 The Rheumatism Research Center, Catholic Research Institute of Medical Science, College of Medicine, The Catholic University of Korea, 222, Banpo-daero, Seocho-gu, Seoul, 06591 South Korea; 2 IMPACT Biotech, Catholic Research Institute of Medical Science, The Catholic University of Korea, Seoul, South Korea; 3 Division of Nephrology, Department of Internal Medicine, Seoul St. Mary’s Hospital, College of Medicine, The Catholic University of Korea, Seoul, South Korea; 4 Divison of Rheumatology, Department of Internal Medicine, Seoul St. Mary’s Hospital, The Catholic University of Korea, 222 Banpo-Daero, Seocho-gu, Seoul, 137-701 South Korea

**Keywords:** Inflammatory bowel disease, Gut microbiota, Probiotics, Fibrosis

## Abstract

**Background:**

An altered gut microbiota balance is involved in the pathogenesis of inflammatory bowel disease (IBD), and several probiotic strains are used as dietary supplements to improve intestinal health. We evaluated the therapeutic effect of 12 probiotics in combination with prebiotics, rosavin, and zinc in the dextran sodium sulfate (DSS)-induced colitis mouse model.

**Methods:**

The probiotic complex or the combination drug was administered orally to mice with DSS-induced colitis, and the body weight, disease activity index, colon length, and histopathological parameters were evaluated. Also, the combination drug was applied to HT-29 epithelial cells, and the expression of monocyte chemoattractant protein 1 (MCP-1) was evaluated by real-time polymerase chain reaction.

**Results:**

Administration of the combination drug attenuated the severity of DSS-induced colitis. Moreover, the combination drug significantly reduced the levels of the proinflammatory cytokines tumor necrosis factor-α, interleukin (IL)-6, IL-1β, and IL-17, and significantly increased the levels of Foxp3 and IL-10 in colon sections. Additionally, treatment with the combination drug reduced MCP-1 expression in HT-29 cells. Treatment with the combination drug decreased the levels of α-smooth muscle actin and type I collagen compared with vehicle treatment in mice with DSS-induced colitis.

**Conclusion:**

These results suggest that the combination of a probiotic complex with rosavin, zinc, and prebiotics exerts a therapeutic effect on IBD by modulating production of pro- and anti-inflammatory cytokines and the development of fibrosis.

## Background

Inflammatory bowel disease (IBD), which comprises Crohn’s disease (CD) and ulcerative colitis (UC), is a chronic, progressive, and destructive inflammatory condition of the gastrointestinal tract caused by multiple genetic and environmental factors [1]. The pathogenesis of IBD is related to a breakdown of intestinal homeostasis, which results in uncontrolled immune responses to the gut microbiota by intestinal epithelial cells and immune cells, leading to complications, such as perforating ulcers and fibrosis [2–5]. Intestinal fibrosis is a common complication and a nonspecific feature of IBD. Increased deposition of collagen causes excessive fibrosis, progressive tissue architectural distortion, dysfunctional wound healing, and luminal narrowing [6, 7].

The interplay between the microbiota and immune cells is important in the pathogenesis of IBD [8]. Interleukin (IL)-17-producing Th17 cells are involved in mucosal immune responses [9, 10]. The IL-17 mRNA level is increased in inflamed mucosa from IBD patients, and the disease severity of IBD is correlated with the IL-17 level in peripheral blood mononuclear cells from UC patients [11, 12]. Furthermore, IL-17 deficiency does not cause colonic inflammation in IBD animal models [13, 14]. T regulatory (Treg) cells play roles that are the opposite of those played by Th17 cells in autoimmune diseases, including in IBD [15]. Treg cells maintain immune-cell homeostasis by suppressing the functions of other immune cell types, particularly Th17 cells. IL-10, which is secreted by Treg cells, is essential for intestinal homeostasis; indeed, IL-10 deficiency leads to the spontaneous development of colitis in mice [16].

Probiotics are live microorganisms that benefit host health when administered in adequate amounts [17]. Probiotics promote maintenance of the gut barrier function and modulation of the host immune system; therefore, dietary supplements containing probiotics may be beneficial for IBD [18–20]. *Lactobacillus*, *Bifidobacterium*, and *Enterococcus* strains are commonly used as probiotics [21–23].

A prebiotic is a non-viable food substance that confers a health benefit on the host by promoting selective growth of beneficial bacteria and is associated with modulation of the intestinal microbiota [24]. A synbiotic, a mixture of a probiotic and prebiotic, has the advantages of both probiotics and prebiotics [25, 26].

In this study, we combined 12 probiotics with prebiotics, rosavin (extracted from *Rhodiola rosea* L.), and zinc. The therapeutic effect of this combination drug in mice with dextran sodium sulfate (DSS)-induced colitis was investigated. The combination drug modulated the production of both proinflammatory cytokines (IL-6, IL-1β, and IL-17) and an anti-inflammatory cytokine (IL-10) in mice with DSS-induced colitis. Furthermore, the combination drug ameliorated intestinal fibrosis.

## Methods

### Mice

Eight-week-old male C57BL/6 mice were purchased from Orient Bio Inc. (Seongnam, Korea). The animals were housed under specific pathogen-free conditions at the Institute of Medical Science of the Catholic University of Korea and were maintained under controlled temperature (21–22 °C) and light (12/12-h light/dark cycle) conditions. The mice were fed standard mouse chow and water. All experimental procedures were approved by the Department of Laboratory Animals, Institutional Animal Care and Use Committee (IACUC) of the School of Medicine, the Catholic University of Korea and conformed with all National Institutes of Health (USA) guidelines (Permit number: CUMC 2016-0244-01).

### Probiotic complex, prebiotics, rosavin and zinc

The probiotic complex (Lot#171121), prebiotics (Chicory fiber, Lot#RCRRW6ARW6), and rosavin (Lot#88843) were purchased from CNS Pharm Korea Co., Ltd (Seoul, Korea). The probiotic complex comprises *Lactobacillus acidophilus*, *Lactobacillus casei*, *Lactobacillus fermentum*, *Lactobacillus paracasei*, *Streptococcous thermophilus*, *Bifidobacterium longum*, *Bifidobacterium bifidum*, *Bifidobacterium breve*, *Lactobacillus rhamnosus*, *Lactobacillus plantarum*, *Lactobacillus helveticus,* and *Lactobacillus salivarius*. The probiotic complex was resuspended in saline at 25 mg/mL and killed by heating at 80 °C for 30 min. Zinc was purchased from Sigma-Aldrich (MO, USA, #205532).

### Induction of colitis and drug administration

C57BL/6 mice were divided into three groups (5 mice in each group) and were group-housed in cage. Groups of mice were administrated 3% DSS (MP Biomedicals, Santa Ana, CA, USA) in drink water. To measure the amount of DSS consumption, DSS-dissolved drinking water (3% DSS) was replaced daily and measure the amount left of drinking water. Each mouse drank about 15 mL of 3% DSS water during the 4 days. Mice were randomly assigned to three groups (n = 5/group) as follows: Vehicle-treated mice were administered DSS in drinking water. Probiotics complex-treated mice were administered the probiotics complex (100 mg/mouse) in drinking water, and combination drug-treated mice were administered the probiotics complex, prebiotics, zinc, and rosavin (100 mg/mouse; probiotics complex [1.25 mg], prebiotics [15 mg], zinc [3 mg], and rosavin [20 mg]) daily beginning 3 days after DSS administration. Body weight and disease activity index (DAI) score were monitored daily. Data are representative of two independent experiments with similar results.

### Assessment of inflammation

The severity of colitis was assessed daily by determining the percentage body weight change and DAI. The DAI was calculated as described previously [27]. Body weight loss (score: 0, none; 1, 0–5%; 2, 6–10%; 3, 11–15%; 4, 16–20%; 5, 21–25%; and 6, 26–30%), stool consistency (score: 0, normal stools; 1, soft stools; and 2, liquid stools), and rectal bleeding (score: 0, negative fecal occult blood; 1, positive fecal occult blood; and 2, visible rectal bleeding) were assessed daily in each mouse.

### Histopathological analysis

Colon tissue Sections (5-μm thick) were fixed and embedded in 10% (v/v) neutral-buffered formalin (Sigma-Aldrich, St. Louis, MO). Histological analysis was performed on H&E stained colitis tissue and scored by two experimenters in a blinded fashion. Stained sections were examined under a photomicroscope (Olympus, Tokyo, Japan) (magnifications: 100×). The histological scoring system was used for evaluation of the degree of colitis [28]. Loss of epithelium, crypt damage, depletion of goblet cells, and infiltration of inflammatory cells were assessed in these sections. Histological evaluation and scoring of loss of epithelium (0, no loss of epithelium; 1, 0–5% loss of epithelium; 2, 5–10% loss of epithelium; and 3, > 10% loss of epithelium), crypt damage (0, no damage; 1, 0–10% loss of crypt; 2, 10–20% loss of crypt; 3, > 20% loss of crypt), depletion of goblet cells (0, none; 1, mild; 2, moderate; and 3, severe), and infiltration of inflammatory cells (0, none; 1, mild; 2, moderate; and 3, severe) were performed. Total histological score were ranged from 0 to 12.

### Immunohistochemistry

Sections were treated with 3% (v/v) H_2_O_2_ in methanol to block endogenous peroxidase activity. Immunohistochemistry was performed using the Envision Detection™ kit (DAKO, Glostrup, Denmark, #5007). Tissue sections were incubated with primary antibodies against IL-10 (Santa Cruz Biotechnology, Santa Cruz, CA, USA, #SC-1783), Foxp3 (Santa Cruz Biotechnology, Santa Cruz, CA, USA, #SC-28705), tumor necrosis factor (TNF)-α (Abcam, Cambridge, UK, #ab6671), IL-1β (Abcam, Cambridge, UK, #ab9722), IL-17 (Abcam, Cambridge, UK, #ab79056), IL-6 (Abcam, Cambridge, UK, #ab7737), α-smooth muscle actin (α-SMA) (Abcam, Cambridge, UK, #ab7817), and type I collagen (Col-I) (Abcam, Cambridge, UK, #ab6308) for 2 h at room temperature. Sections were then incubated with a horseradish peroxidase (HRP)-conjugated secondary antibody for 30 min. The final colored products were developed using chromogen diaminobenzidine, and the sections were examined under a photomicroscope (Olympus, Tokyo, Japan). Positive cells were enumerated visually by four individuals, and the mean values were calculated.

### Stimulation of HT-29 cells

HT-29 cells were seeded in 24-well plates at an initial density of 5 × 10^4^ cells per well and allowed to adhere for 12 h. HT-29 cells were maintained in RPMI-1640 supplemented with 10% fetal bovine serum. After 12 h, the medium was replaced by a fresh one and the cells were pretreated with combination drug for 2 h and then the cells were incubated in the presence of lipopolysaccharide (LPS; 1 μg/mL, Sigma-Aldrich) for 2 days. The supernatants were assayed for monocyte chemoattractant protein 1 (MCP-1) levels.

### Enzyme-linked immunosorbent assay

The levels of MCP-1 in HT-29 cell culture supernatants were measured by sandwich enzyme-linked immunosorbent assay (ELISA; R&D Systems). Horseradish peroxidase–avidin (R&D Systems) was used for color development. Absorbance at 405 nm was measured using an ELISA microplate reader (Molecular Devices, Sunnyvale, CA, USA).

### Statistical analysis

All statistical analyses were performed using GraphPad Prism (version 4 for Windows; GraphPad Software). Normally distributed continuous data were analyzed using the parametric Student’s *t* test. Differences in mean values among groups were subjected to analysis of variance (ANOVA) followed by Bonferroni post hoc test. Data are presented as means ± standard deviations (SDs). A value of *p* < 0.05 (two-tailed) was considered to indicate statistical significance.

## Results

### Effect of the combination drug on DSS-induced colitis

**Fig. 1:**
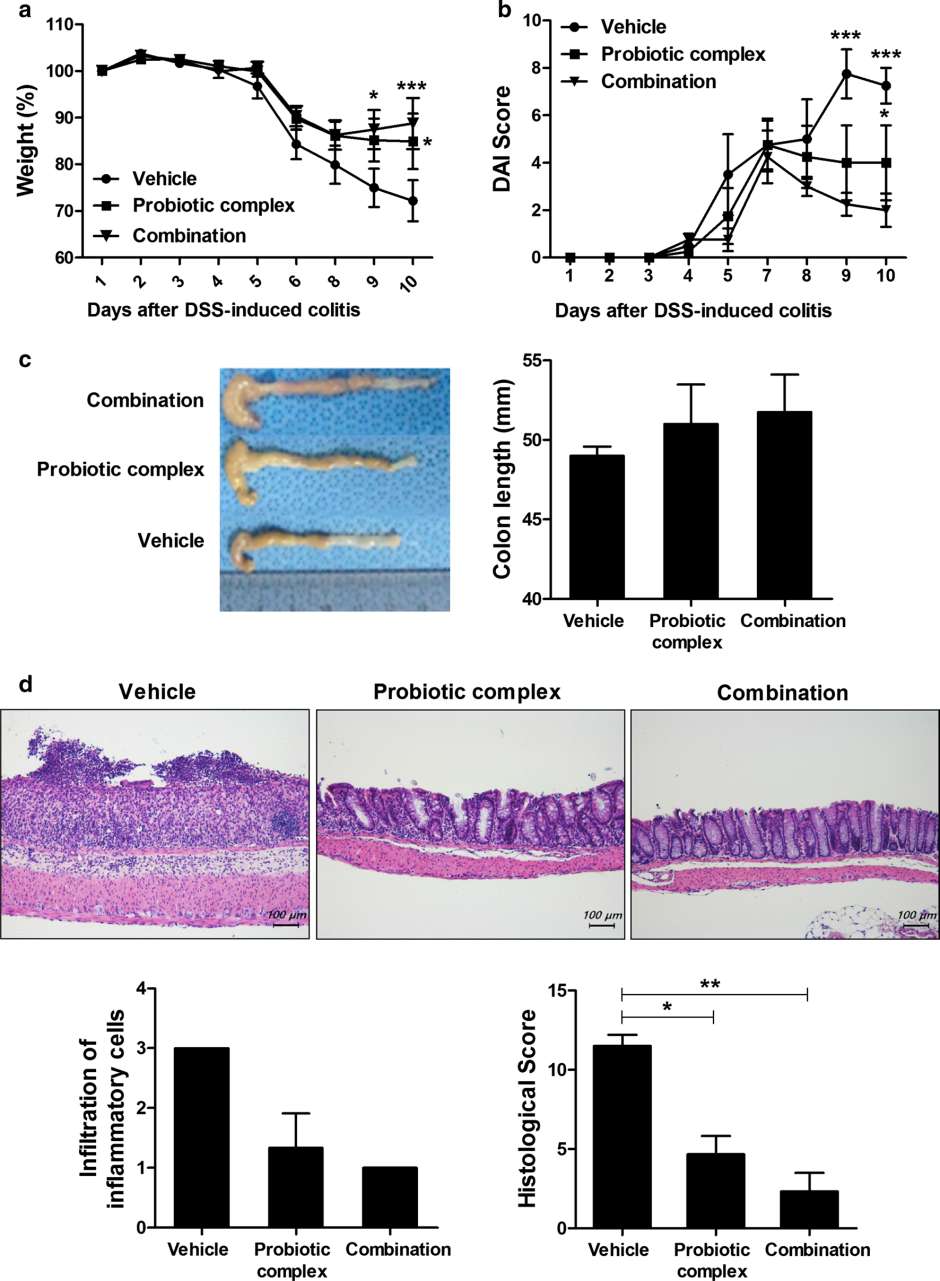
The combination drug ameliorated DSS-induced colitis. C57BL/6 mice were treated orally with 3% DSS in distilled water ad libitum from days 0 to 4 and thereafter received regular drinking water. On day 3 after DSS administration, the probiotic complex or the combination drug (100 mg/mouse) was administered orally (n = 5/group). **a** Changes in body weight expressed as percentages of body weight on day 0. **b** With the exception of day 7, the DAI was monitored daily. Data are means ± SDs of four independent experiments. **c** Colon length of mice with DSS-induced chronic colitis on day 10. Macroscopic images of the colon (left panel). Colon length (right panel). **d** Representative hematoxylin and eosin-stained images of the colon on day 10. Scale bar, 100 µm. ^*^p < 0.05, ^**^p < 0.01, ^***^p < 0.001. Vehicle *vs*. probiotic complex or combination drug treatment

To investigate the therapeutic effects of probiotics complex and the combination drug, the probiotics complex and the combination drug in drinking water were administered to mice daily after DSS administration. Mice in the vehicle-treated DSS-induced colitis group showed marked weight loss from day 5 as a result of severe colitis (Fig. 1a). However, treatment with the probiotic complex (p < 0.05) or the combination drug (p < 0.001) ameliorated the loss of body weight. The DAI score, which combines weight loss, changes in stool consistency, and bleeding, was significantly lower in the probiotic complex—(p < 0.05) and combination drug-treated groups (p < 0.001) compared to the vehicle-treated group (Fig. 1b). Treatment with the probiotic complex or the combination drug prevented the decrease in colon length evident in the vehicle-treated group (Fig. 1c). Microscopic examination of the colon revealed that the probiotic complex and the combination drug restored the mucosal architecture compared with vehicle treatment. The histological score was decreased in the probiotic complex—(p < 0.05) and combination drug-treated groups (p < 0.01) (Fig. 1d). The combination drug exerted a greater therapeutic effect than did the probiotic complex.

### Effect of the combination drug on proinflammatory cytokine and chemokine expression

**Fig. 2:**
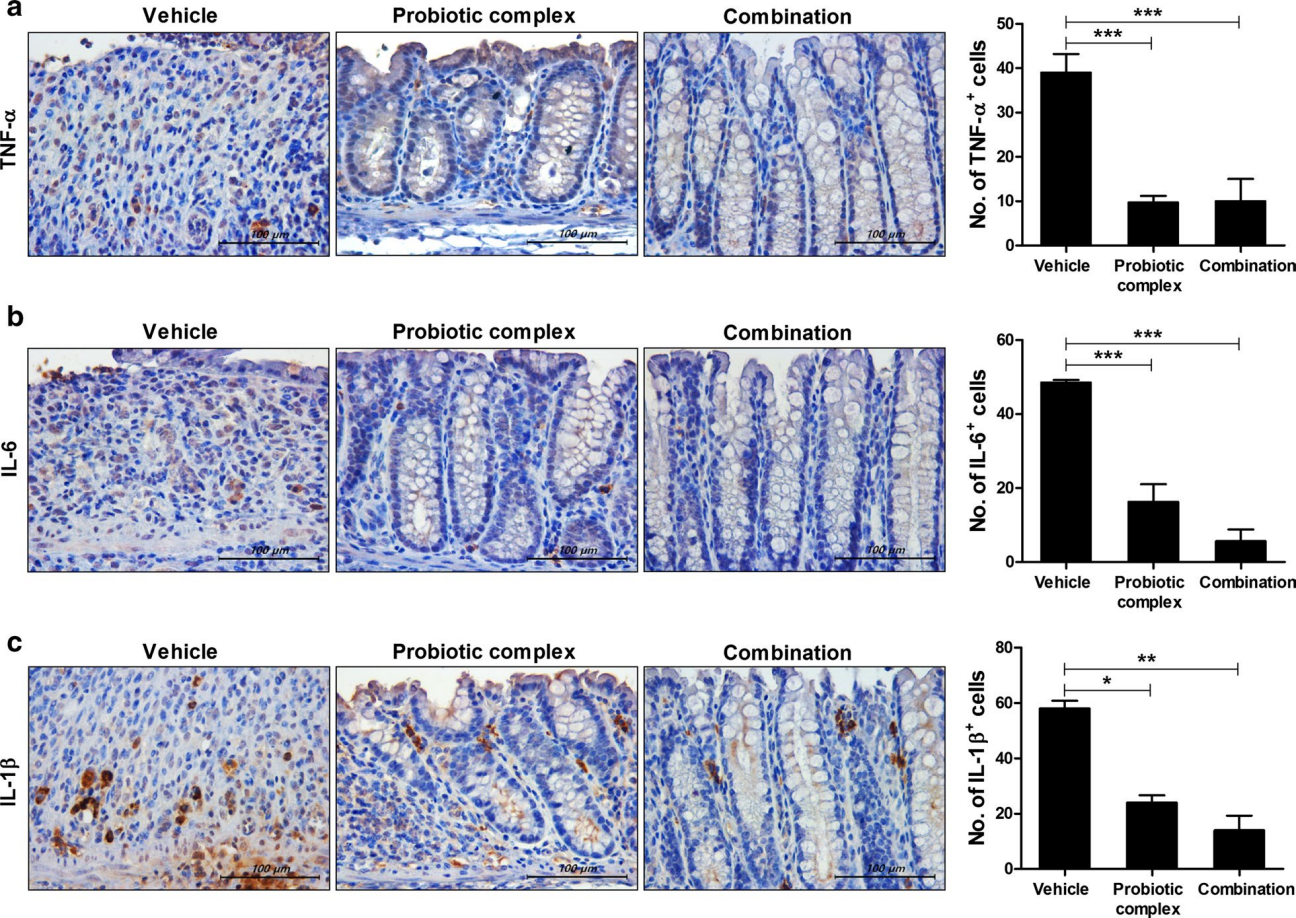
The combination drug reduced proinflammatory cytokine levels. C57BL/6 mice were treated orally with 3% DSS in distilled water ad libitum from days 0 to 4 and thereafter received regular drinking water. On day 3 after DSS administration, the probiotic complex or the combination drug (100 mg/mouse) was administered orally (n = 5/group). On day 10 after DSS administration, colon tissue sections were stained with antibodies against TNF-α (**a**), IL-6 (**b**), and IL-1β (**c**). Representative histological features are shown, and the results are presented as mean ± SD numbers of antibody-positive cells (n = 5/group). Scale bar, 100 µm. ^*^p < 0.05, ^**^p < 0.01, ^***^p < 0.001. Vehicle vs. probiotic complex or combination drug treatment

**Fig. 3:**
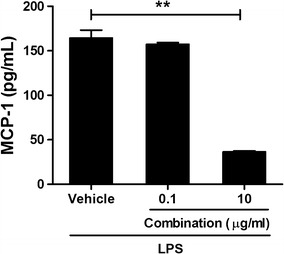
The combination drug reduced the MCP-1 level in HT-29 cells. HT-29 cells were treated with the combination drug in the presence of LPS (1 μg/mL) for 48 h. MCP-1 mRNA and protein levels were determined by RT-PCR and ELISA, respectively. ^**^p < 0.01 by Student’s *t* test. Vehicle vs. combination drug treatment

The effect of the probiotic complex and the combination drug on colitis was assessed by immunostaining colon sections for inflammatory cytokines (Fig. 2). Colon tissue from vehicle-treated mice had higher levels of TNF-α, IL-6, and IL-1β than did colon tissue from mice treated with the probiotic complex or the combination drug; the latter exerted the greatest effect on colitis (p < 0.001, p < 0.001, and p < 0.01, respectively). To assess the immune modulatory effects of combination drug, the HT-29 cell line, a human colorectal adenocarcinoma cell line, was pretreated with combination drug for 2 h and then incubated in the presence of LPS for 48 h. The level of MCP-1, which regulates migration and infiltration of monocytes/macrophages [29], in HT-29 cells was decreased by treatment with the combination drug (p < 0.01) (Fig. 3).

### Effect of the combination drug on IL-17, Foxp3, and IL-10 levels

**Fig. 4:**
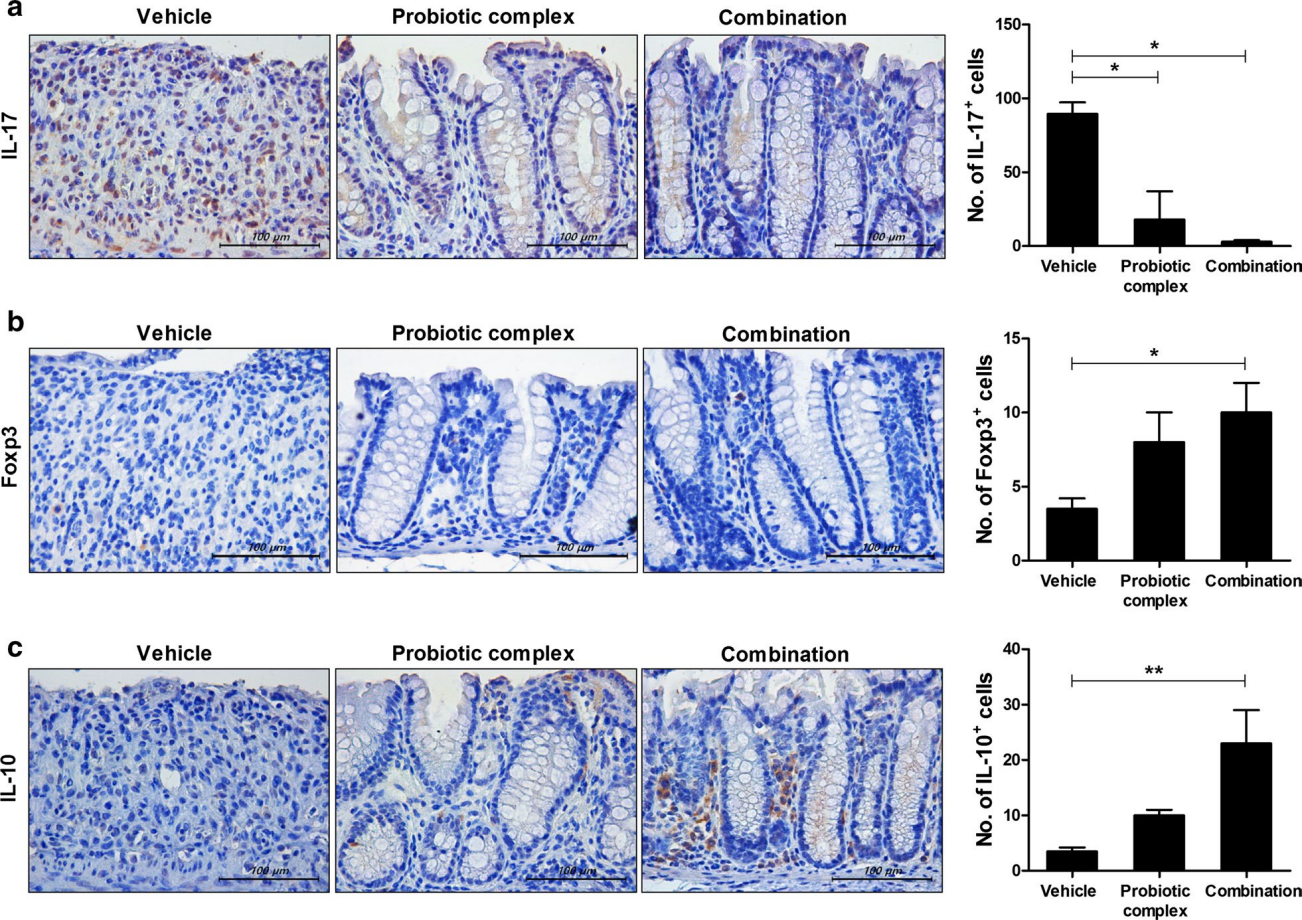
The combination drug reduces IL-17 and Foxp3 levels. C57BL/6 mice were treated orally with 3% DSS in distilled water ad libitum from days 0 to 4 and thereafter received regular drinking water. On day 3 after DSS administration, the probiotic complex or the combination drug (100 mg/mouse) was administered orally (n = 5/group). On day 10 after DSS administration, colon tissue sections were stained with antibodies against IL-17 (**a**), Foxp3 (**b**), and IL-10 (**c**). Representative histological features are shown, and the results are presented as mean ± SD numbers of antibody-positive cells (n = 5/group). Scale bar, 100 µm. ^*^p < 0.05, ^**^p < 0.01. Vehicle vs. probiotic complex or combination drug treatment

Th17 cells infiltrate the inflamed intestine, where they trigger and amplify inflammation [8]. To investigate the effect of the probiotic complex and the combination drug on IL-17, Foxp3 (a transcription factor for Treg cells [30]), and IL-10 levels, colon sections were subjected to immunohistochemical staining. The colon tissue of mice treated with the probiotic complex or the combination drug exhibited a lower IL-17 level than did the colon tissue of vehicle-treated mice (p < 0.05) (Fig. 4a). In contrast, Foxp3 and IL-10 levels were significantly increased in the colon tissue of mice treated with the combination drug compared to those treated with vehicle (p < 0.05 and p < 0.001, respectively) (Fig. 4b, c). Treatment with the probiotic complex also increased the expression of Foxp3 and IL-10, albeit not significantly so.

### Effect of the combination drug on colitis-associated intestinal fibrosis

**Fig. 5:**
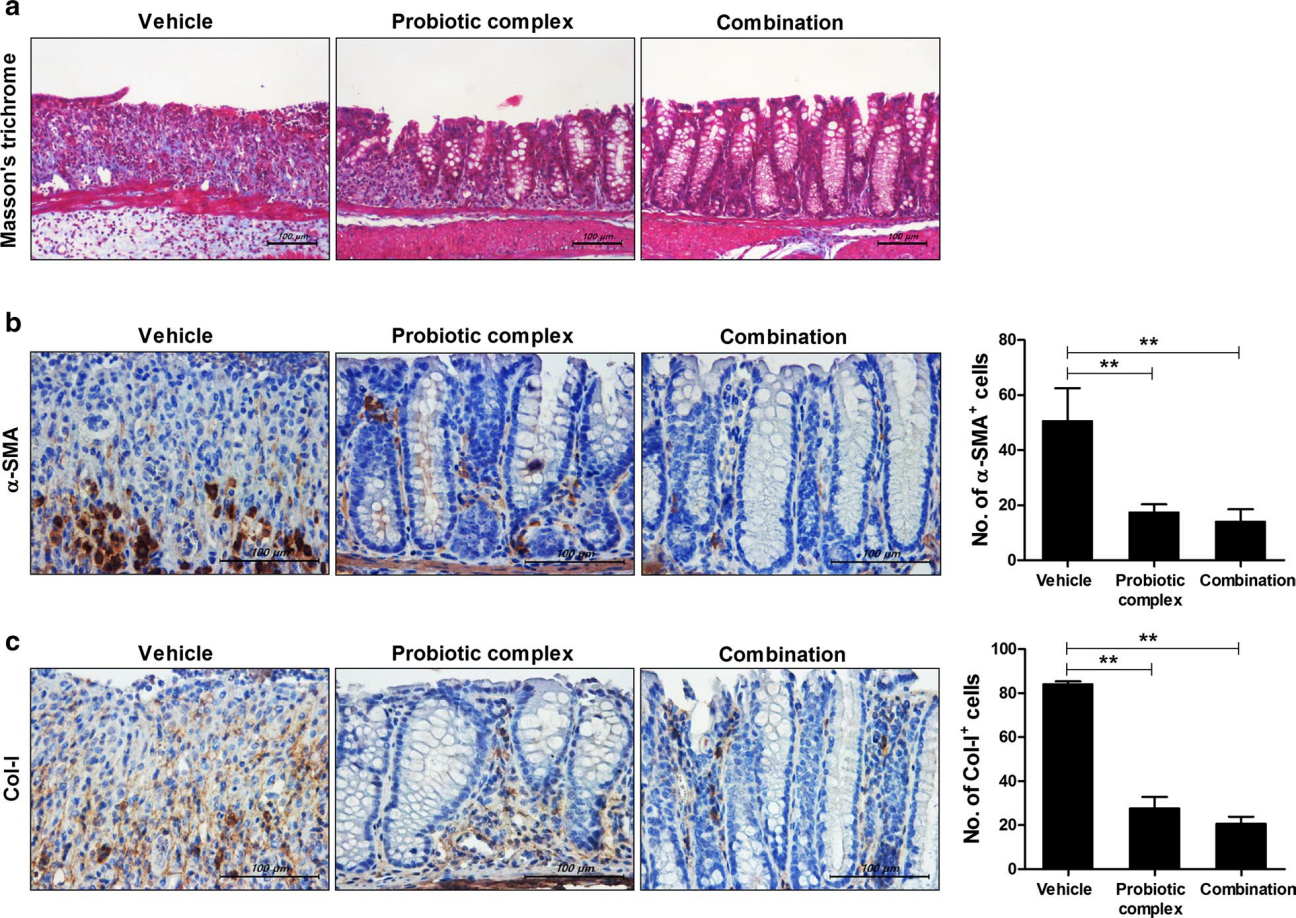
The combination drug reduces colitis-associated intestinal fibrosis. C57BL/6 mice were treated orally with 3% DSS in distilled water ad libitum from days 0 to 4 and thereafter received regular drinking water. On day 3 after DSS administration, the probiotic complex or the combination drug (100 mg/mouse) was administered orally (n = 5/group). At 10 days after DSS administration, colon tissue sections were stained with Masson’s trichrome (**a**). Representative histological features are shown. **b**, **c** Colon tissue was subjected to immunohistochemical staining for α-SMA (**b**) and Col-I (**c**). Representative histological features are shown, and the results are presented as mean ± SD numbers of antibody-positive cells (n = 5/group). ^**^p < 0.01. Vehicle vs. probiotic complex or combination drug treatment

To investigate the antifibrotic effects of the probiotic complex and combination drug, colon sections were stained with Masson’s trichrome (Fig. 5a). Treatment with the probiotic complex or the combination drug suppressed the area of blue staining in the colonic muscular layer compared with vehicle treatment. To evaluate the effect of the probiotic complex or the combination drug on the number of myofibroblasts in the colonic mucosa and submucosa, immunohistochemical staining for α-SMA, a marker of fibroblast differentiation to myofibroblasts, was performed on day 10. The number of α-SMA+ myofibroblasts was significantly decreased in the colons of mice treated with the probiotic complex or the combination drug compared with the vehicle-treated mice (p < 0.01) (Fig. 5b). Furthermore, the number of Col-1+ cells was significantly decreased in the colons of mice treated with the probiotic complex or the combination drug compared with vehicle-treated mice (p < 0.01) (Fig. 5c).

## Discussion

The aim of this study was to elucidate the effect of a combination of a probiotic complex, prebiotics, rosavin, and zinc in a DSS-induced colitis mouse model. Treatment with the combination drug attenuated the severity of colitis; suppressed the body weight loss, reduction in colon length and DAI score; and reduced the histological changes evident in vehicle-treated mice. The combination drug decreased the levels of proinflammatory cytokines and chemokines and increased the levels of Foxp3 and IL-10 in colon tissue. Furthermore, treatment with the combination drug suppressed colitis-associated intestinal fibrosis.

Gut microbiota dysbiosis plays a key role in the pathogenesis of IBD. Probiotics, live microorganisms that benefit host health, are commonly used to control chronic gastrointestinal inflammation in IBD patients. *Lactobacillus* and *Bifidobacterium* species are widely used as probiotics [31]. Administration of *Lactobacillus plantarum* LP ameliorates the severity of colitis in IL-10-knockout mice [32]. *Lactobacillus paracasei* LS2 isolated from the Korean food, kimchi, reduces colitis disease by decreasing the number of Th1 cells and macrophages in the lamina propria [33]. Treatment with *Lactobacillus acidophilus* reduces the STAT3 and phosphorylated STAT3 levels in colon tissue from mice with DSS-induced colitis and increases the number of Treg cells among intestinal intraepithelial and lamina propria lymphocytes in a 2,4,6-trinitrobenzene sulfonic acid-induced colitis model [21, 34]. Treatment with *Bifidobacterium longum* ameliorates colorectal colitis in rats by altering the methylation level of the Foxp3 promoter, resulting in an increased number of Treg cells [35]. Moreover, *Streptococcus thermophilus* suppresses bacterial translocation, which reduces gastrointestinal bleeding and weight loss [36].

Much research has focused on the use of gut microbiota for the treatment of IBD. Treatment with lactobacilli and bifidobacteria promoted recovery of DSS-induced intestinal injury and inflammation in a mouse model of colitis [37]. *Lactobacillus casei* and *Bifidobacterium lactis* ameliorated injury to the intestinal mucosa and liver in a 2,4,6-trinitrobenzene sulfonic acid-induced colitis model [38]. Also, treatment with a probiotic combination that included lactobacilli, bifidobacteria, and streptococci reduced the levels of proinflammatory cytokines in colitis [39]. In this study, we evaluated the efficacy of a combination of 12 probiotics (lactobacilli, bifidobacteria, and streptococci) with prebiotics, rosavin, and zinc for the treatment of IBD. *Rhodiola rosea* is a widely used plant for traditional medicine and it was reported that it has anti-oxidant, neuroprotective, anti-diabetic and anti-inflammatory effects [40–42]. Rosavin is a typical compound of *Rhodiola rosea* and recent study revealed that it inhibits TNF-related apoptosis-inducing ligand expression via extracellular signal-regulated kinase phosphorylation in T cells [43]. Zinc is absorbed in the small intestine, functions as a cofactor for enzymes, such as alkaline phosphatase, and is essential for growth, immunity, and tissue repair [44, 45]. Furthermore, zinc involves in the regulation of cell proliferation via affecting metalloenzymes and hormonal signaling. Reduced zinc availability influences survival of animal [46]. Zinc deficiency is commonly seen in IBD patients due to inadequate zinc intake or poor absorption from the gastrointestinal tract [45, 47, 48]. In the present study, oral administration of the combination drug showed therapeutic effects in a DSS-induced colitis model. Notably, the combination drug resulted in a greater decrease in the levels of proinflammatory cytokines (TNF-α, IL-6, IL-1β, and IL-17) and greater increases in the levels of the anti-inflammatory cytokine IL-10 and transcription factor Foxp3 in colon tissue compared with the probiotic complex alone. Further studies should investigate the mechanisms underlying the effect of the combination drug on intestinal homeostasis.

The combination drug also improved DSS-induced intestinal fibrosis. Fibrosis develops from chronic inflammation and is caused by the accumulation of extracellular matrix components, which causes stricture formation and organ dysfunction [6, 7]. Fibrosis is a major and frequent complication in IBD patients, for which no specific therapy is available [49]. The combination drug decreased the α-SMA and type I collagen levels compared with vehicle treatment. Recently, there have been attempts to treat fibrosis using cytokine-targeted therapy. IL-10 deficiency accelerates kidney inflammation and fibrosis in the unilateral ureteral obstruction mouse model, and treatment with IL-10 ameliorates lung fibrosis [50, 51]. In contrast, IL-17 exerts pro-fibrotic effects in the lung, liver, and heart [52]. IL-17 deficiency reduces fibrosis in models of skin inflammation, and treatment with IL-17 enhances cardiac fibroblast proliferation and migration in pulmonary fibrosis models [53, 54].

DSS-induced colitis model used in this study is very popular for the IBD research because of its rapidity, simplicity, reproducibility and controllability. However, the limitation of this model is that T or B cell responses are not required for the development of colitis unlike human disease [55]. In addition, luminal bacteria play a role in the development of this robust colitis [56]. Therefore, additional studies are needed to determine the complex role of combination of a probiotic complex with rosavin, zinc, and prebiotics on the gut-immune axis.

## Conclusion

In conclusion, treatment with the combination of a probiotic complex, prebiotics, rosavin, and zinc attenuated colitis, significantly decreased the levels of proinflammatory cytokines, and significantly increased the levels of Foxp3 and IL-10 in colon tissue. Furthermore, treatment with the combination drug decreased the levels of α-SMA and Col-I compared with vehicle treatment. Therefore, the combination of a probiotic complex with rosavin, zinc, and prebiotics may be important therapeutics for IBD by modulating the inflammatory cytokines and fibrosis.

## Abbreviations

IBD: inflammatory bowel disease
CD: Crohn’s disease
UC: ulcerative colitis
IL: interleukin
Treg: T regulatory
DAI: disease activity index
TNF: tumor necrosis factor
α-SMA: α-smooth muscle actin
Col-I: type I collagen
LPS: lipopolysaccharide
MCP-1: monocyte chemoattractant protein 1
ELISA: enzyme-linked immunosorbent assay
